# The Involvement of MicroRNAs in Osteoarthritis and Recent Developments: A Narrative Review

**DOI:** 10.31138/mjr.29.2.67

**Published:** 2018-06-29

**Authors:** Panagiotis K. Panagopoulos, George I. Lambrou

**Affiliations:** 1 Postgraduate Program “*Metabolic Bone Diseases*”, National and Kapodistrian University of Athens, Medical School, Athens, Greece; 2 First Department of Pediatrics, National and Kapodistrian University of Athens, Choremeio Research Laboratory, Athens, Greece

**Keywords:** miRNAs, osteoarthritis, epigenetics, dysregulation

## Abstract

**Background:**

Osteoarthritis (OA) is the most common chronic joint disease and it may progressively cause disability and compromise quality of life. Lately, the role of miRNAs in the pathogenesis of OA has drawn a lot of attention. miRNAs are small, single-stranded, non-coding molecules of RNA which regulate gene expression at post-transcriptional level. The dysregulation of the expression of several miRNAs affects pathways involved in OA pathogenesis.

**Objective:**

The purpose of this article is to review the literature on the involvement of miRNAs in the pathogenesis of OA and the implications on its diagnosis and treatment.

**Materials and Methods:**

An extensive electronic literature search was conducted by two researchers from January 2008 to August 2017. Titles and abstracts of papers were screened by the authors for further inclusion in the present work. Finally, full texts of the selected articles were retrieved.

**Results:**

Abnormally expressed miRNAs enhance the production of cartilage degrading enzymes, inhibit the expression of cartilage matrix components, increase the production of proinflammatory cytokines, facilitate chondrocyte apoptosis, suppress autophagy in chondrocytes and are involved in pain-related pathways. miRNAs are also incorporated in extra-cellular membranous vesicles such as exosomes and participate in the intercellular communication in osteoarthritic joints.

**Conclusion:**

Ongoing research on miRNAs has potential implications in the diagnosis and treatment of OA. Their different levels in peripheral blood and synovial fluid between OA patients and healthy population makes them candidates for being used as biomarkers of the disease, while targeting miRNAs may be a novel therapeutic strategy in OA.

## INTRODUCTION

Osteoarthritis (OA) is the most common chronic arthropathy and is characterised by failure of damaged cartilage to repair itself, synovial inflammation and changes in the subchondral bone. Increased production of cartilage-degrading enzymes (Matrix Metalloproteinases, aggrecanases) by articular chondrocytes, insufficient synthesis of cartilage matrix components (collagen type II, aggrecan) and increased chondrocyte apoptosis lead to gradual cartilage loss. Pain and stiffness are the main clinical features of OA. Loss of movement and function are features of more severe disease, resulting in a worse quality of life.^1^ The etiology of OA is complex and not fully understood yet. It involves genetic and environmental factors, such as joint injury, obesity and aging.^2^

According to epidemiological and family-based genetic studies, genetic factors seem to be responsible for a significant proportion of OA susceptibility. Heritability has been estimated to be 39–79% depending on the affected joint, gender and severity of diseases. In addition, these studies have shown that OA is a complex polygenic disorder – multiple risk loci contribute to OA heritability, each of which accounts for a small proportion of it.^3^ During the last decade, large Genome Wide Association Studies (GWAS) have identified 17 genetic loci for OA,^4–14^ but these risk loci do not fully account for OA heritability. Epigenetic modifications may be responsible for OA heritability that remains unexplained by OA genetics. Epigenetics include heritable mechanisms, such as DNA methylation, histone modifications and microRNAs, which regulate gene expression without changes to the DNA sequences.^15–16^ MicroRNAs (miRNAs) have attracted a lot of attention lately, since they have the potential to be used as biomarkers or as novel therapeutic agents. Numerous studies have shown that miRNA expression is altered in OA and these alterations possibly contribute to OA pathogenesis. The purpose of this article is to review the literature on the role of miRNAs in the pathogenesis of OA and its implications on diagnosis and treatment of this disorder.

### MicroRNAs

**Figure 1.**
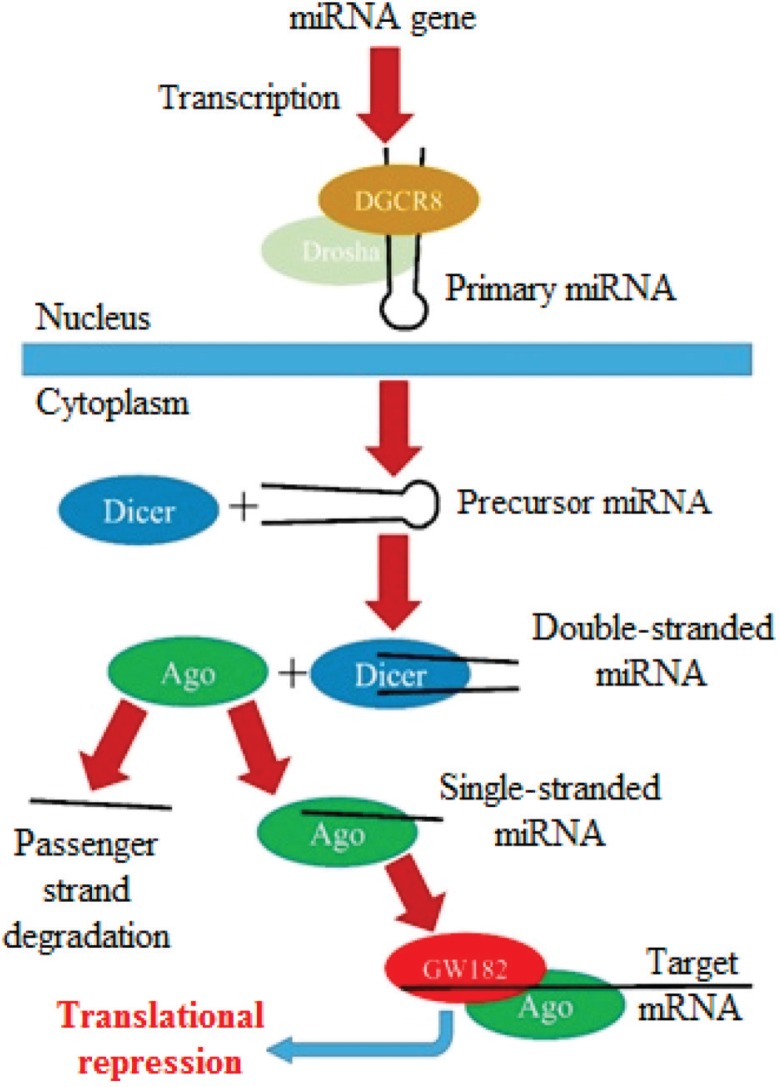
The synthesis of miRNAs and their mechanism of action (adjusted from Miao et al. 2013) (Miao, Yang et al. 2013). *Figure 1 has been redesigned and created from scratch based on the work of Miao et al. 2013, using the Microsoft Office ® PowerPoint software*.^20^

MicroRNAs (miRNAs) are small, single-stranded, non-coding RNAs, consisting of 20–25 nucleotides, whose role is post-transcriptional regulation of gene expression. MiRNAs are partially complementary and bind to the 3′-Untranslated Region (3′-UTR) of their target messenger RNA (mRNA). They then inhibit the translation of their target mRNA or cause its degradation. Thus, miRNAs inhibit the expression of their target gene at post-transcriptional level.^17^ Concerning the synthesis of miRNAs, primary miRNA is transcribed in the nucleus from its gene (**Figure 1**). Afterwards, ribonuclease Drosha and protein DGCR8 process the primary miRNA to precursor miRNA. The precursor miRNA is transferred to the cytoplasm and ribonuclease Dicer processes it to mature miRNA. The passenger strand of the miRNA is ejected and degraded and the other strand – the mature miRNA – is loaded to protein Argonaute (Ago). The mature miRNA interacts with proteins Ago and GW182, binds to its target mRNA and inhibits its translation.^18^ miRNAs are encoded by DNA sequences which are found in the genome either as separate miRNA genes or within the introns of other genes. Over 3% of human genes have been found to contain miRNA-coding sequences, while the expression of 40–90% of human protein-coding genes is regulated by miRNAs.^19^ The expression of a protein-coding gene may be regulated by more than one miRNA and each miRNA may regulate the expression of several target genes.^17^ miRNAs participate in many biological procedures, such as cell differentiation, proliferation and apoptosis, and they are involved in several diseases, including cancer, viral infections and autoimmune diseases.^18^

Apart from binding to their target mRNAs and inhibiting their translation, miRNAs may be packaged and transferred extracellularly by three different ways: (a) incorporated in extracellular membranous vesicles (exosomes, shedding vesicles and apoptotic vesicles), (b) bound to lipoproteins, like High Density Lipoprotein (HDL), and (c) bound to RNA-binding proteins, like Argonaute-2 and nucleophosmin-1. These miRNAs are secreted via exocytosis, they may be received by other cells via endocytosis and regulate their gene expression. Thus, miRNAs participate in intercellular communication.^20–21^

### The Role of MicroRNAs in OA

A remarkable number of studies have been published during the last few years about the expression of different miRNAs in osteoarthritic cartilage and subchondral bone. Most of these studies examine the expression of miRNAs targeting genes known to participate in the pathogenesis of OA. For this purpose, we have summarized in **Table 1** the miRNAs found to be dysregulated in OA and their target genes.

**Table 1:** MicroRNAs dysregulated in OA and their target genes.

**MicroRNA**	**Target gene**	**Reference**
miR-9	MCPIP1	Makki, Haseeb et al. 2015^54^
	PTRG	Song, Kim, Chun et al. 2013^80^
miR-15a-5p	VEGFA	Chen et al. 2017^81^
miR-16-5p	*SMAD3*	Li L et al. 2015^48^
miR-21	*GDF5*	Zhang et al. 2014^82^
	*GAS5*	Song et al. 2014^60^
miR-23a-3p	*SMAD3*	Kang et al. 2016^47^
miR-24	*INK4A*	Philipot et al. 2014^39^
miR-26a	iNOS	Rasheed et al. 2016^36^
	KPNA3	Yin et al. 2017^34^
miR-26b	KPNA3	Yin et al. 2017^34^
miR-27b	*MMP13*	Akhtar et al. 2010^37^
miR-29	Smad, NFκB, and canonical Wnt signaling	Le et al. 2016^83^
miR-30a	*ADAMTS5*	Ji, Xu, Zhang et al. 2016^45^
miR-30b	ERG	Li, Yang et al. 2015^84^
miR-33	CCL2	Wei et al. 2016^85^
miR-33a	*SMAD7*	Kostopoulou et al. 2015^86^
mir-34a	SIRT1	Yan et al. 2016^56^
miR-98	-	Wang GL et al. 2016^87^
miR-105	Runx2	Ji, Xu, Xu et al. 2016^44^
miR-122	*IL1A*	Yang et al. 2015^88^
miR-125b	*ADAMTS4*	Matsukawa et al. 2013^46^
miR-127	OPN	Tu et al. 2016^89^
miR-130	TNFA	Li ZC et al. 2015^51^
miR-139	MCPIP1	Makki and Haqqi, 2015^53^
	EIF4G2, IGF1R	Hu et al. 2016^90^
miR-140	*ADAMTS5*	Miyaki et al. 2009,^22^ Miyaki et al. 2010^29^
	*IGFBP5*	Tardif et al. 2009^25^
	*MMP13*	Liang et al. 2012,^24^ Liang et al. 2016^91^
miR-142-3p	HMGB1	Wang X et al. 2016^49^
miR-146	*MMP13*	Yamasaki et al. 2009^30^
	*MMP13, ADAMTS5*	Li et al. 2011^31^
	*SMAD4*	Li et al. 2012^33^
miR-148	*COL10A1*, MMP13, ADAMTS5	Vonk et al. 2014^43^
miR-149	*TNFA*, *IL1B*, *IL6*	Santini et al. 2014^52^
miR-155	*ULK1*, *MAP1LC3*, *ATG14*	D’ Adamo et al. 2016^59^
miR-181	PTEN	Wu et al. 2017^92^
miR-210	*DR6*	Zhang et al. 2015^50^
	HIF-3α	Li Z et al. 2016^38^
miR-222	HDAC-4	Song, Jin et al. 2015^40^
miR-335	-	Tornero-Esteban et al. 2014^93^
miR-370	SHMT-2	Song, Kim et al. 2015^41^
miR-373	MECP-2	Song, Kim et al. 2015^41^
miR-381a-3p	IkBα	Xia et al. 2016^55^
miR-483-5p	*Matn3*, *Timp2*	Wang et al. 2017^77^
miR-488	*ZIP8*	Song, Kim, Lee et al. 2013^42^
miR-558	*COX2*	Park et al. 2013^94^
miR-634	PIK3R1	Cui et al. 2016^95^

Each microRNA is reported with its respective target genes and the bibliographical reference where the information was obtained.

### miR-140 in OA

**Figure 2.**
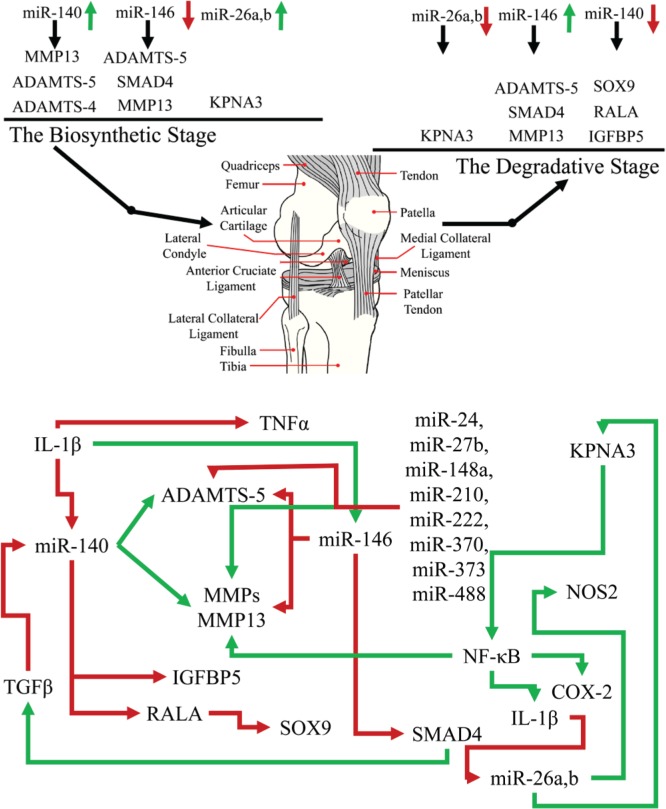
Schematic representation of basic miRNAs and their target genes. It is interesting that similar miRNAs could manifest a stimulatory as well as an inhibitory role in OA. Depending on their expression levels miRNAs play a respective role, either as OA stimulatory or OA inhibitory factors. In the lower sub-figure, the basic interactions are presented between miRNAs and their target genes (**Legend:** in the upper sub-figure, green arrows indicate up-regulation and red arrows indicate down-regulation. In the lower sub-figure, green arrows indicate stimulation or activation and red arrows indicate inhibition).

One of the most studied miRNAs in OA is miRNA-140 (miR-140). The expression of miR-140 in chondrocytes increases during their differentiation, suggesting that it is probably a regulator of the differentiation of these cells. In osteoarthritic cartilage the expression of miR-140 is reduced in comparison to healthy cartilage.^22–23^ Target genes of miR-140 include ADAMTS5 (ADAM Metallopeptidase with Thrombospondin Type 1 Motif 5), MMP13 (Matrix Metalloproteinase 13), IGFBP5 (Insulin Like Growth Factor Binding Protein 5) and RALA (RAS like proto-oncogene A).^22,24–26^ ADAMTS5 and MMP13 are proteinases that mediate the degradation of several components of cartilage matrix and might play an important role in OA pathogenesis.^22,24^ IGFBP-5 (Insulin-like Growth Factor Binding Protein 5) is also involved in OA pathology by modulating the availability of IGF-1 in the joint.^25^ RALA (RAS like proto-oncogene A) is a small GTPase that downregulates the transcription factor SOX9 (SRY-box 9). SOX9 is a master regulator of cartilage development and it enhances the production of cartilage matrix components. Downregulation of RALA by miR-140 results in upregulation of SOX9.^26^ Concerning the expression of miR-140, the cytokine Interleukin-1β (IL-1β), a key player in OA pathogenesis, inhibits the expression of miR-140 by chondrocytes,^22,24^ while the transcription factor SOX9 enhances its expression (27). Moreover, the transcription factor SMAD3 (SMAD family member 3), a mediator of Transforming Growth Factor-β (TGF-β), downregulates miR-140 expression by articular chondrocytes.^28^ Therefore, Interleukin-1β (IL-1β) and Transforming Growth Factor-β (TGF-β) inhibit the expression of miR-140 in chondrocytes of osteoarthritic cartilage, resulting in increased expression of ADAMTS5, MMP13, IGFBP5 and RALA and degradation of articular cartilage matrix.^22,24–26,28^ In addition, targeted deletion of miR-140 in mice resulted to OA-like changes of articular cartilage, while overexpression of miR-140 in cartilage protected it from antigen-induced arthritis, enhancing the hypothesis of miR-140 participating in OA pathogenesis.^29^ The basic interactions of miR-140 and its target genes is presented in **Figure 2**.

### miR-146 in OA

Another miRNA that has been studied in OA is miR-146. Its expression is increased in osteoarthritic cartilage during the early stages of the disease and it gradually decreases as OA progresses. The target gene of miR-146 is MMP13 and the expression of this miRNA is upregulated by IL-1β. Thus, it seems that miR-146 is a negative feedback regulator of MMP13 and it possibly plays a protective role in OA cartilage.^30^ Indeed, miR-146 inhibits IL-1β-induced MMP13 and ADAMTS5 production by chondrocytes and IL-1β-induced suppression of collagen type II and aggrecan, which are components of the cartilage matrix.^31^ miR-146 also inhibits IL-1β-induced TNF-α upregulation in OA cartilage.^32^ Moreover, miR-146 downregulates the expression of SMAD4, a transcription factor that is a mediator of TGF-β. Thus, upregulation of miR-146 in OA chondrocytes downregulates SMAD4, reduces cellular responsiveness to TGF-β and induces chondrocyte apoptosis. Downregulation of SMAD4 also leads to an increase in the expression of Vascular Endothelial Growth Factor (VEGF), which contributes to inflammation and pathological angiogenesis in OA.^33^ Furthermore, altered expression of miR-146 appears to play a role in pain-related pathways in OA. miR-146 is downregulated in dorsal root ganglia and in the dorsal horn of the spinal cords of rats with osteoarthritic pain. miR-146 decreases the expression of pain modulators that enhance pain perception, such as Tumor Necrosis Factor-α (TNF-α), Interleukin-6 (IL-6), Interleukin-8 (IL-8), COX-2 and iNOS, in astrocytes. Thus, it seems that downregulation of miR-146 in the central and peripheral nervous system of the rat OA model mediates osteoarthritic pain.^31^ The basic interactions of miR-146 and its target genes is presented in **Figure 2**.

### miR-26a and miR-26b in OA

The role of miR-26a and miR-26b in the pathogenesis of OA has been recently studied. The expression of miR-26a and miR-26b is significantly downregulated in cartilage from osteoarthritic joints, while the target gene of these miRNAs has been found to be the one encoding Karyopherin Subunit Alpha 3 (KPNA3). KPNA3 is a mediator of Nuclear Factor-κB (NF-κB) pathway which binds to NF-κB and facilitates its translocation from cytoplasm to nucleus.^34^ It is suggested that the NF-κB pathway might play a significant role in OA pathogenesis, since it induces production of proinflammatory cytokines, Cyclooxygenase-2 (COX-2) and metalloproteinases (MMPs), which result in joint inflammation and degradation of joint cartilage.^34,35^ Therefore, downregulation of miR-26a and miR-26b in OA cartilage results in upregulation of KPNA3 and NF-κB and production of MMPs and COX-2.^34^ In addition, activation of NF-κB pathway negatively regulates the expression of miR-26a, implying a reciprocal inhibition between miR-26a and NF-κB. Moreover, obesity, a well known risk factor of OA, induces the activation of NF-κB, resulting in downregulation of miR-26a expression.^35^ On the other hand, another target gene of miR-26a is NOS2, which encodes inducible Nitric Oxide Synthase (iNOS). In OA, activation of iNOS in chondrocytes results in Nitric Oxide (NO) overproduction leading to chondrocyte apoptosis, cartilage degradation and inhibition of matrix synthesis. Thus, IL-1β downregulates miR-26a expression in OA chondrocytes through NF-κB activation, resulting in upregulation of iNOS expression, overproduction of NO and cartilage damage.^36^

### MicroRNAs involved in cartilage matrix degradation and joint inflammation in OA

Dysregulation of the expression of several miRNAs in OA results in increased production of cartilage matrix degrading enzymes (MMPs, ADAMTS proteases). Downregulation of miR-24, miR-27b, miR-148a, miR-210, miR-222, miR-370, miR-373 and miR-488 in OA cartilage leads directly or indirectly to an increase in the production of MMPs,^37–43^ while downregulation of miR-30a, miR-105, miR-125b and miR-148a results in overproduction of ADAMTS proteases.^43–46^ Moreover, upregulation of miR-16-5p and miR-23a-3p leads to upregulation of MMPs and ADAMTS proteases and downregulation of matrix components (type II collagen, aggrecan).^47–48^

Besides, other miRNAs are involved in the production of proinflammatory cytokines like TNF-α, IL-1, IL-6 and IL-8 in OA. Downregulation of miR-142-3p and miR-210 in chondrocytes of OA cartilage leads to overexpression of High Mobility Group Box 1 (HMGB1) and Death Receptor 6 (DR6) respectively. As a result, the NF-κB signaling pathway is activated and the production of TNF-α, IL-1 and IL-6 is increased.^49,50^ Downregulation of miR-130a and miR-149 in OA chondrocytes results also in an increase in the production of TNF-α, IL-1 and IL-6.^51–52^ Upregulation of miR-9 and miR-139 in chondrocytes of OA cartilage downregulates the expression of Monocyte Chemoattractant Protein–Induced Protein 1 (MCPIP-1), thus promoting IL-6 expression and the apoptosis of chondrocytes.^53,54^ Moreover, upregulation of miR-381a-3p in OA chondrocytes inhibits IκBα (nuclear factor of kappa light polypeptide gene enhancer in B-cells inhibitor, alpha), resulting in an increase of the production of TNF-α, COX-2, iNOS, IL-6 and IL8.^55^

### MicroRNAs involved in apoptosis in OA

Aberrant expression of miRNAs facilitates the apoptosis of articular chondrocytes, thus enhancing the degradation of articular cartilage. The involvement of miR-9,^54^ miR-26a,^36^ miR-139^53^ and miR-146^33^ in increased apoptosis of chondrocytes in osteoarthritic cartilage has already been discussed. Another miRNA involved in chondrocytes apoptosis in OA is miR-34a. The expression of miR-34a is upregulated in human articular chondrocytes isolated from OA patients. The target gene of miR-34a encodes Silent Information Regulator 1 (SIRT1), a deacetylase playing a crucial role in the prevention of cell apoptosis. The upregulation of miR-34a in osteoarthritic chondrocytes results in downregulation of SIRT1 leading to upregulation of pro-apoptotic protein Bax (BCL2 associated X, apoptosis regulator), downregulation of anti-apoptotic protein Bcl-2 (B cell leukemia/lymphoma 2) and promotion of cell apoptosis.^56^

miR-210 is also involved in increased apoptosis of osteoarthritic chondrocytes. The expression of miR-210 is inhibited in OA chondrocytes, resulting to upregulation of its target gene DR6. Increased expression of DR6 leads to increased activation of the NF-κB signaling pathway and facilitates the apoptosis of the chondrocytes.^50^ In addition, miR-222 is downregulated in OA chondrocytes, resulting in increased expression of Histone Deacetylase 4 (HDAC-4) and increased cell apoptosis.^40^ Besides, miR-195 is overexpressed in peripheral blood of patients with OA.^57^ The target gene of miR-195 encodes Hypoxia-Inducible Factor 1 alpha (HIF-1α). *In vitro* study in chondrocyte cell cultures has shown that overexpression of miR-195 results in downregulation of HIF-1α and in increased apoptosis of chondrocytes.^58^

### MicroRNAs and autophagy in OA

Autophagy is a cell response to stress, in which cytoplasmic organelles and macromolecules are degraded by lysosomes and then recycled in order to support cellular metabolism and survival. Aging and age-related diseases, including OA, are related to reduced autophagy. Lately, several studies have been published about the involvement of microRNAs in reduced autophagy in OA.^59–61^

miR-155 is upregulated in human osteoarthritic cartilage and takes part in reduced autophagy in OA chondrocytes. Bioinformatics predict that miR-155 targets the autophagy-related genes ATG3 (autophagy related 3), GABARAPL1 (GABA type A receptor associated protein-like 1), ATG5 (autophagy related 5), ATG2B (autophagy related 2B), LAMP2 (lysosomal associated membrane protein 2) and FOXO3 (forkhead box O3). Recent *in vitro* study confirmed that miR-155 downregulates the expression of ATG3, GABARAPL1, ATG5 and FOXO3 in human articular chondrocytes, as well as the expression of other autophagy-related genes (ULK1 [unc-51-like autophagy activating kinase 1], MAP1LC3 [microtubule-associated protein 1 light chain 3 beta] and ATG14 [autophagy-related 14]), resulting in inhibition of autophagy. D’ Adamo et al. conclude that miR-155 inhibits autophagy in chondrocytes and is partially responsible for defective autophagy in OA.^59^

miR-21 is another miRNA whose dysregulated expression leads to decreased autophagy in OA. Its target gene is GAS5 (Growth arrest-specific 5), which stimulates cell apoptosis and suppresses autophagy. The expression of miR-21 is decreased in osteoarthritic chondrocytes, resulting in upregulation of GAS5, increased apoptosis and suppressed autophagy. Besides, GAS5 downregulates miR-21, implying a reciprocal interplay between miR-21 and GAS5. Furthermore, when miR-21 was injected in osteoarthritic joints of a mouse OA model, it reduced cartilage destruction, whereas intra-articular injection of an inhibitor of miR-21 worsened cartilage destruction.^60^ On the other hand, increased expression of miR-146 seems to have a protective effect in osteoarthritic cartilage by promoting chondrocytes autophagy. Zhang et al. studied the effect of hypoxia, a pathogenetic mechanism contributing to OA development, on the expression of miR-146 and autophagy in chondrocytes. They demonstrated that hypoxia induces HIF-1α (Hypoxia-inducible factor-1α) in chondrocytes, which upregulates the expression of miR-146a. Upregulated miR-146a suppresses Bcl-2, an autophagy inhibitor, resulting in promotion of autophagy. Zhang et al. conclude that miR-146a plays probably a protective role in OA by enhancing chondrocyte autophagy.^61^

### Profiling multiple microRNAs expressed in osteoarthritic tissues

The aforementioned studies have examined the expression of one or a few miRNAs, which target a gene or a pathway that is already known to participate in the pathogenesis of OA. On the other hand, during recent years, other studies have used high-throughput methods, such as hybridization microarrays and next generation RNA-sequencing, in order to examine the profile of multiple miRNAs expressed in the cartilage and subchondral bone of osteoarthritic joints and compare it to healthy controls.^32,62–68^ A summary of the respective studies is presented in **Table 2**. Although these studies have some results in common, such as the downregulation of miR-140, most of their results do not overlap. There are plenty of reasons for this variety of results. Some studies measured miRNA expression in fresh samples of cartilage, subchondral bone or synovial fluid from osteoarthritic joints, while other studies used cultured chondrocytes from OA cartilage. Moreover, different studies used different sets of microarrays in order to examine the miRNA expression, while one study used next generation RNA-sequencing. In addition, sample size was small and there were no adjustments for confounding factors, such as age, gender or obesity. However, these studies have identified a lot of new miRNAs and genes that potentially participate in OA pathogenesis. Further studies will investigate the role of these miRNAs in OA and reveal novel pathogenetic mechanisms related with them.

**Table 2.** High-throughput methods used in OA literature.

**Study**	**Experimental material**	**Number of samples**	**Methodology**	**Results**
Jones et al. 2009^32^	Cartilage and subchondral bone from OA vs normal joints	4/4	Microarrays (157 miRNAs)	47 differentially expressed miRNAs
Iliopoulos et al. 2008^64^	Cultured chondrocytes from OA vs normal cartilage	33/10	Microarrays (365 miRNAs)	11 differentially expressed miRNAs
Swingler et al. 2012^67^	Discovery: Cultured chondrocytes Validation: Cartilage from OA vs normal joints	10/10	Discovery: Microarrays Validation: RT-PCR	39 miRNAs differentially expressed during chondrogenesis 2 miRNAs differentially expressed in OA vs normal cartilage
Diaz-Prado et al. 2012^63^	Cultured chondrocytes from OA vs normal cartilage	6/4	Microarrays (723 miRNAs)	7 differentially expressed miRNAs
Tornero-Estaban et al. 2015^68^	Cultures of bone marrow mesenchymal stem cells from OA patients vs controls	10/10	Microarrays (754 miRNAs)	246 differentially expressed miRNAs
Crowe et al. 2016^62^	Discovery: Cartilage from OA joints Validation: Cartilage from OA vs normal joints	11/6	Discovery: Next generation RNA-sequencing	60 new miRNAs expressed in OA cartilage 3 differentially expressed
Li YH et al. 2016^65^	Synovial fluid from late-stage vs early-stage OA joints	4/4	Validation: RT-PCR Microarrays (752 miRNAs)	miRNAs 7 differentially expressed miRNAs
Rasheed et al. 2016^66^	Cultured chondrocytes from OA cartilage, stimulated or not with IL-1β	Unknown	Microarrays (1347 miRNAs)	36 differentially expressed miRNAs

Studies that used high-throughput methods in order to examine the profile of multiple miRNAs expressed in cartilage, subchondral bone and synovial fluid of osteoarthritic joints.

### Extracellular Vesicles and microRNAs IN OA

miRNAs may be packaged in extracellular vesicles such as exosomes, secreted from the cell that produces them and transferred to another cell, regulating thus the gene expression of the latter.^20^ In OA, miRNAs in exosomes are altered and these alterations seem to get involved in OA pathogenesis. Recent study demonstrated that the expression of several miRNAs was altered in exosomes contained in synovial fluid derived from osteoarthritic joints compared to normal joints.^69^ In another study, Kato et al. used IL-1β to stimulate synovial fibroblasts and examined the effect of exosomes derived from the stimulated synovial fibroblasts on articular chondrocytes. IL-1β is a key player of OA pathogenesis mediating synovial inflammation and cartilage degradation. Kato et al. demonstrated that exosomes from IL-1β-stimulated synovial fibroblasts upregulated the expression of degrading enzymes MMP13 and ADAMTS5 in articular chondrocytes and downregulated the expression of cartilage matrix components (type II collagen and aggrecan). They also showed that the expression of 50 miRNAs was dys-regulated in exosomes derived from IL-1β-stimulated synovial fibroblasts compared with non-stimulated synovial fibroblasts.^70^ In addition, Nakasa et al. showed that exosomes derived from IL-1β-stimulated OA cartilage up-regulated the expression of MMP13, IL-1β, TNF-α and COX-2 in OA synovium.^71^ Thus, miRNAs packaged in exosomes participate in OA pathogenesis by mediating cell to cell communication in osteoarthritic joints.

### MicroRNAs as Biomarkers in OA

miRNAs may be detected in peripheral blood and syno-vial fluid incorporated in extracellular vesicles or bound to lipoproteins and RNA-binding proteins.^20,21^ The stability of miRNAs in circulation^72^ and their different levels between patients with OA and healthy population offer the opportunity of using these molecules as biomarkers for this disease. Murata et al. showed that plasma levels of miR-16 and miR-132 differentiated OA patients from healthy controls, since they were significantly lower in the former. Moreover, synovial fluid concentrations of miR-16, miR-146a, miR-155 and miR-223 were significantly lower in patients with OA compared to patients with rheumatoid arthritis and could differentiate those two groups of patients. In the same study, Murata et al. discovered that there was no correlation between plasma and synovial fluid miRNA levels, implying different origins for them, and then demonstrated that synovial membrane is the main source of synovial fluid miRNAs.^72^ In another study, Borgonio Cuadra et al. compared plasma levels of 380 miRNAs between OA patients and healthy subjects and found 12 miRNAs that were overexpressed in the plasma of OA patients (miR-16, miR-20b, miR-29c, miR-30b, miR-93, miR-126, miR-146a, miR-184, miR-186, miR-195, miR-345, miR-885-5p).^57^ Recently, Withrow et al. demonstrated that the concentration of miR-7-5p and miR-200c-3p in exosomes derived from synovial fluid was significantly higher in OA patients in comparison to healthy subjects.^69^ Moreover, Okuhara et al. have shown that peripheral blood mononuclear cells express significantly higher levels of miR-146a, -155, -181a, and -223 in OA patients compared to healthy population.^73^ Furthermore, in an interesting study, Beyer et al. investigated the possibility of using plasma miRNA levels in order to predict the development of severe knee and hip OA. They discovered that lower plasma levels of let-7e were associated with severe knee and hip OA requiring total joint arthroplasty.^74^ Therefore, although results are limited and sometimes contradicting, miRNAs have the potential of being used as biomarkers for OA. Their stability, ease of measurement and different expression in the blood and synovial fluid of OA patients offer the opportunity of using them to predict the prognosis or even measure disease activity or predict response to treatment. However, more studies are needed for this to become possible.

### Therapeutic Potential of microRNAs in OA

Current treatment of OA includes drugs such as Nonsteroidal Anti-Inflammatory Drugs (NSAIDS) for alleviating symptoms and total joint arthroplasty in cases of severe OA. There are no drugs that halt the progress of the disease, like disease-modifying drugs do in rheumatoid arthritis.^1^ MiRNAs represent a promising target for the treatment of OA. A remarkable number of miRNAs participate in the pathogenesis of OA. Inhibition of these miRNAs with antisense oligonucleotides (anti-miRs) or administration of miRNAs that silence genes participating in OA pathogenesis could be a novel approach for arresting the progress of OA. An advantage of this approach is that synovial joints are an isolated environment and intra-articular administration of miRNAs would not have systemic effects. However, an important issue is the delivery method of the miRNAs or the anti-miRs. Several solutions have been proposed, including extracellular vesicles (exosomes), nanoparticles and antibodies.^75^

An example of targeting miRNAs for the treatment of OA is the inhibition of miR-34a. The upregulation of miR-34a in osteoarthritic chondrocytes results in inhibition of SIRT1, leading to increased cell apoptosis.^56^ Abouheif et al. demonstrated that silencing of miR-34a with oligonucleotides of antisense miR-34a inhibited chondrocytes apoptosis in rat chondrocyte cultures treated with IL-1β.^76^ Yan et al. recently examined the results of suppressing miR-34a in rats with OA. Oligonucleotides of antisense miR-34a (anti-miR-34a) were cloned into a lentivirus vector and the lentiviral vectors were injected into the osteoarthritic joints of the rats. Intra-articular injection of lentiviruses encoding anti-miR-34a ameliorated cartilage destruction of the OA joints.^56^

miR-483-5p is upregulated in articular cartilage from OA patients and it targets and downregulates matrilin 3 (Matn3) and tissue inhibitor of metalloproteinase 2 (Timp2). Matn3 is a protein of the cartilage matrix and Timp2 is an inhibitor of cartilage degrading metalloproteinases. Wang et al. recently studied the results of silencing miR-483-5p in an experimental OA mouse model. Lentiviruses encoding oligonucleotides of antisense miR-483-5p (anti-miR-483-5p) were injected in the OA joints and it was demonstrated that anti-miR-384-5p attenuated cartilage damage and loss and inhibited the formation of fibrous cartilage.^77^

miR-140 is one of the most studied miRNAs in OA. Karlsen et al. studied the protective effect of miR-140 in an *in vitro* model of OA. They transfected miR-140 into IL-1β-treated articular chondrocyte and mesenchymal stem cell cultures and they demonstrated that miR-140 upregulated the synthesis of cartilage matrix components and downregulated the production of cartilage degradation enzymes.^78^ In a recent study, Tao et al. used exosomes in order to transfer miR-140 into osteoarthritic joints in a rat OA model. They acquired exosomes rich in miR-140 by transfecting mesenchymal stem cells (MSCs) with lentivirus encoding miR-140 and by isolating the exosomes derived from the miR-140-overexpressing-MSCs. They first transfected articular chondrocytes with miR-140-exosomes and showed that miR-140 downregulated RALA and upregulated SOX9, aggrecan and collagen type II. Then they injected miR-140-exosomes into osteoarthritic joints of rats and demonstrated that miR-140 reduced the damage of the articular cartilage in comparison to the control group.^79^

## CONCLUSIONS

Multiple studies demonstrate that miRNAs potentially play an important role in the pathogenesis of OA. The dysregulation of their expression affects several pathways involved in OA pathogenesis. Dysregulated miRNAs increase the expression of cartilage degrading enzymes by articular chondrocytes, decrease the production of cartilage matrix components, facilitate chondrocyte apoptosis and inhibit autophagy in chondrocytes, thus contributing to cartilage damage. They are also involved in the production of proinflammatory cytokines and the induction of joint inflammation, as well as in pain-related pathways in OA. In addition, miRNAs are incorporated in extracellular membranous vesicles such as exosomes and transferred from one cell to another. Thus, they participate in the communication between synoviocytes and articular chondrocytes in osteoarthritic joints, enhancing the production of degrading enzymes and cytokines by these cells.

The role of miRNAs in OA still remains to be elucidated. Yet, based on the available data and the overall role of miRNA molecular machinery, it is possible to gain some insight on their participation in OA. Hence, there are some general concepts governing miRNA physiology. Their role depends mainly on the target gene. This means that if a gene has an enhancing or suppressive effect on a certain physiological procedure, the down- or up-regulation of the respective miRNA signifies the opposite effect. For example, in the case of miR-146 we have mentioned that it decreases the expression of pain modulators that enhance pain perception, such as TNF-α, IL-6, IL-8, COX-2 and iNOS, in astrocytes. Thus, it seems that *downregulation* of miR-146 in the central and peripheral nervous system signals the *upregulation* of the target genes. In other words, the genes that mediate pain are inhibited by miR-146. Further on, although it is known that miRNA expression and binding to target-genes is linked to gene negative regulation, the only way to determine miRNA role is through experimental validation; this varies from one pathophysiological condition to another.

Besides, it seems that dysregulation of miRNA expression in OA is both a consequence of upstream events (such as increased production of proinflammatory cytokines) and a consequence of negative feedback from downstream events. For example, as mentioned above, increased production of the proinflammatory cytokine IL-1*β* in OA results in the downregulation of miR-26a and miR-140. On the other hand, downregulation of miR-26a in OA leads to upregulation of KPNA3 and activation of NF-κB pathway. In turn, the activated NF-κB pathway negatively regulates miR-26a (negative feedback).

Moreover, the protective or harmful role of miRNA in OA is a subject of intensive discussion. As aforementioned, several miRNAs have been reported to have a protective role in OA, such as miR-140 or miR-146, yet at the same time several other miRNAs are reported to play a negative role in OA, such as miR-155 and miR-195.

On the other hand, different expression of miRNAs in peripheral blood and synovial fluid between OA patients and healthy population, their stability in body fluids and the ease of their measurement creates the potential of utilizing them as biomarkers of the disease. Besides, next generation RNA-sequencing will facilitate the identification of new miRNAs in order to be used as biomarkers. In the future, miRNAs may be used as biomarkers of disease activity or as predictors of prognosis or of response to treatment. However, further studies are still needed in this direction.

Furthermore, targeting of miRNAs is a potential novel therapeutic strategy in OA. Inhibition of miRNAs contributing to OA pathogenesis or administration of miRNAs silencing genes participating in OA pathogenesis has been studied in animal OA models. Intra-articular injection of anti-miRs and of miRNAs has been proved successful in animals. However, delivery of these molecules in OA joints remains an issue. Exosomes may be an option for OA treatment, but further research is necessary.

## LIST OF ABBREVIATIONS

**Table:**

**Abbreviation**	**Explanation**
miRNAs	Micro RNAs
Osteoarthritis	OA
mRNA	Messenger RNA
3′-UTR	3′-untranslated region
MMPs	Metalloproteinases
NSAIDS	Nonsteroidal anti-inflammatory drugs
MMPs	Matrix Metalloproteinases
GWAS	Genome Wide Association Studies
3′-UTR	3′-Untranslated Region
HDL	High Density Lipoprotein
TGF-β	Transforming Growth Factor-β
RALA	RAS like proto-oncogene A
SOX9	SRY-box 9
SMAD3	SMAD family member 3
ADAMTS5	ADAM Metallopeptidase with Thrombospondin Type 1 Motif 5
MMP13	Matrix Metalloproteinase 13
IGFBP5	Insulin Like Growth Factor Binding Protein 5
RALA	RAS like proto-oncogene A
VEGF	Vascular Endothelial Growth Factor
SMAD3	SMAD family member 3
TGF-β	Transforming Growth Factor-β
KPNA3	Karyopherin Subunit Alpha 3
NF-κB	Nuclear Factor-κB
iNOS	Nitric Oxide Synthase
NO	Nitric Oxide
HMGB1	High Mobility Group Box 1
DR6	Death Receptor 6
TNF-α	Tumor Necrosis Factor alpha
IL-8	Interleukin 8
IL-6	Interleukin 6
MCPIP-1	Monocyte Chemoattractant Protein–Induced Protein 1
IκBα	nuclear factor of kappa light polypeptide gene enhancer in B-cells inhibitor, alpha
SIRT1	Silent Information Regulator 1
HDAC-4	Histone Deacetylase 4
HIF-1α	Hypoxia-Inducible Factor 1 alpha
Bax	BCL2 associated X, apoptosis regulator
Bcl-2	B cell leukemia/lymphoma 2
ATG3	autophagy related 3
ATG14	autophagy related 14
MAP1LC3	microtubule-associated protein 1 light chain 3 beta
ULK1	unc-51 like autophagy activating kinase 1
GABARAPL1	GABA type A receptor associated protein like 1
ATG5	autophagy related 5
ATG2B	autophagy related 2B
LAMP2	lysosomal associated membrane protein 2
FOXO3	forkhead box O3
GAS5	Growth arrest-specific 5
NSAIDS	Nonsteroidal Anti-Inflammatory Drugs
